# EPHect – the Endometriosis Phenome (and Biobanking) Harmonisation Project – may be very helpful for clinicians and the women they are treating

**DOI:** 10.12688/f1000research.9850.1

**Published:** 2017-01-05

**Authors:** Laura M. Miller, Neil P. Johnson

**Affiliations:** 1 Fertility Plus, Reproductive Endocrinology and Fertility Unit, National Women’s, Greenlane Clinical Centre, Auckland, New Zealand; 2 Robinson Research Institute, University of Adelaide, Adelaide, Australia

**Keywords:** Endometriosis, Phenome Harmonisation Project, research, clinical history, laparoscopy

## Abstract

This article acts as a summary of the recently published papers by the World Endometriosis Research Foundation aiming to set up the Endometriosis Phenome and Biobanking Harmonisation Project.  The objective of this project is to standardise recording of patient history and characteristics, recording of surgical procedure and extent of disease as well as collection, processing and storage of specimens and consequently create a reliable resource for research into endometriosis.

The World Endometriosis Research Foundation (WERF) set up the Endometriosis Phenome and Biobanking Harmonisation Project (EPHect) which has recently published four papers ^1– 4^ aimed at the standardisation of reporting and pathological processing. This consensus was reached after two workshops in 2013 covering all four topics (surgical phenotype; ^1^ clinical and covariate phenotype; ^2^ fluid biospecimen collection, processing and storage; ^3^ tissue collection, processing and storage ^4^) with 54 leaders in endometriosis research, sampling and management worldwide.

EPHect is, as far as we are aware, unprecedented in its attempt to provide standards to endeavour to harmonise phenotypic data collection and biological sampling protocols for a specific disease. It remains to be seen whether the initiative will truly attain its objective of facilitating global collaborative research in endometriosis, but the ball is now in the court of national leaders in endometriosis research to ensure that this unique opportunity is not missed.

EPHect is primarily designed to “facilitate large-scale internationally collaborative, longitudinal, epidemiologically robust, translational, biomarker and treatment target discovery research in endometriosis” (a more noble endeavour we cannot envisage – and we couldn’t have put it better ourselves!). Although not specifically designed (nor even intended) for such, we believe that this also presents an opportunity for clinical leaders and clinicians even in a purely clinical setting, to harmonise the collection of standardised clinical and co-variate phenotype data ^1, 2^. Doing so will mean that we gradually become more fluent in a common endometriosis language and more versed in a true understanding of what has to date appeared a heterogeneous disease, not uncommonly described as an enigma ^1, 2^. This level of standardisation of documentation will surely be just as important in the future for optimising patient-focused individualised care as for research. And familiarity with the standard documentation will facilitate clinical leaders to engage in collaborative research efforts seamlessly.

Certainly the collection of “standardized detailed information” and “thus optimizing the surgical phenotype” ^1^ should become a non-negotiable standard each time surgery is undertaken for endometriosis, as the recognition dawns that only a small percentage of women suffering from endometriosis worldwide will ever have the chance to undergo surgery. It is thus imperative that as much information as possible is gained each time a woman undergoes laparoscopic surgery for endometriosis and that this information is comparable with any other woman in the world having surgery for endometriosis.

Two surgical data forms were created ^1^. The standard (recommended) surgical form (EPHect SSF) and the minimum required surgical form (EPHect MSF). We believe that ‘surgeons with expertise’ (relating to the ‘networks of expertise’ that the World Endometriosis Society consensus group on current management of endometriosis has previously described ^5^) should collect information to complete the SSF. The EPHect SSF has two parts. The first asks for clinical covariates (details of clinical relevance) such as last menstrual period, current medical therapy and previous endometriosis surgery and findings. The second part focuses on intraoperative findings such as duration of procedure, extent of endometriosis (location, size and colour of endometriotic lesions, as well as surgical treatment undertaken), location of biopsies, intraoperative complications, extent of residual endometriosis at the end of the surgery and any other pathological findings. Standardisation of the way in which we undertake surgery is a feature that we have now identified to be lacking in terms of diagnosis and classification of endometriosis ^5^. Thus this EPHect initiative should produce a standardised way in which we all undertake laparoscopy and laparoscopic removal of endometriosis. Specifically, the EPHect laparoscopic surgical technique calls for:

- Meticulous search of the entire pelvis and abdominal cavity with a “close tip technique” (2–5 cm distance between laparoscope tip and peritoneal surface)
- Limited handling of the peritoneum during the diagnostic phase of the laparoscopy to minimise petechiae formation
- Video documentation of exploration and the surgical procedure if possible
- Photographic documentation of surgery is considered an acceptable standard and we encourage all endometriosis surgeons to familiarise themselves with the standardised photograph zones (the pelvis is split into seven zones, three in the midline and two for each side, and photographs should be taken with the laparoscope 5–10 cm from the peritoneum). If small lesions or extra-pelvic lesions are present or more detail is deemed appropriate than additional pictures may be required for full documentation. Another additional photograph of the pelvis at the end of the procedure should document any residual disease.

**Table:** 

ZONE	BOUNDARIES	CONTENTS
I	Bilateral: round ligaments. Posterior: uterus. Midline anterior abdominal cavity limited by the round ligaments bilaterally and the uterus posteriorly.	Midline anterior abdominal peritoneum. Anterior surface of the broad ligaments. Bladder dome. Internal ring and inferior epigastric vessels.
II	Anterior: bladder peritoneum. Bilateral: broad ligaments and adnexae. Caudal: torus uterinus	Uterus.
III	Anterior: torus uterinus. Bilateral: uterosacral ligaments. Posterior: sacral pelvic brim	Pouch-of-Douglas/posterior cul-de-sac. Rectovaginal septum. Sigmoid colon. Presacral peritoneum.
IV	Anterior: right round ligament Medial: right broad ligament and adnexe.	Right lateral peritoneum. Anterior surface of right broad ligament.
V	Medial: uterus and right uterosacral ligament. Anterior: right fallopian tube. Lateral: right infundibulopelvic ligament.	Fallopian tube and ovary. Mesovarium. Posterior surface of the broad ligament. Ovarian fossa. Vessels and ureter.
VI	Anterior: left round ligament. Medial: left broad ligament and adnexe.	Left lateral peritoneum. Anterior surface of left broad ligament.
VII	Medial: uterus and left uterosacral ligament. Anterior: left fallopian tube. Lateral: left infundibulopelvic ligament.	Fallopian tube and ovary. Mesovarium. Posterior surface of the broad ligament. Ovarian fossa. Vessels and ureter.
VIII	N/A	Other abdominal pelvic area including: adipose, bowel, ureter, anterior abdominal wall
IX	N/A	Site outside abdominal pelvic area including: umbilicus, scars, chest

- Documentation of other pathology such as adhesions, scarring and uterine fibroids.
- Where feasible, the use of electric or light energy should be avoided in removing tissue samples, as these may cause artifacts that may impact on the histological interpretation of tissue samples – and all energy sources used for this purpose should be recorded. The type of thermal energy recommended, if required surgically, is laser or plasma jet, and, in these circumstances, an excision margin of 5 mm is recommended.
- Extent of residual endometriosis at the end of the procedure should be described.
- The temperature of carbon dioxide insufflation gas entering the peritoneal cavity and the presence or absence of a dehumidifier should be recorded.
- When sampling of different tissues is required, the sequence of sampling should be the order of priority, with the most important tissue or tissue of greatest interest being sampled first.

Two clinical and covariate phenotype data forms have also been created in a similar fashion to the surgical phenotype data: a standard endometriosis patient questionnaire (EPQ-S) and the minimum required endometriosis patient questionnaire (EPG-M) ^2^. These clinical data, again, are geared towards research, however clinicians must seriously consider moving towards routine clinical data collection in order to manage their patients optimally and to determine how research data translates to their own patient population and to individual women. The EPQ-S includes questions on the following:

1. *Pain* – Quantified on 11 point scale for intensity (0 being no pain to 10 meaning worse imaginable pain). Pain affect is captured with the short form McGill Pain Questionnaire (SF-MPQ) ^6^. However, it is recommend to use the most recent SF-MPQ -2 as this again uses an 11 point scale and has 7 additional questions and so would allow for calculations on a total of 4 domains (continuous pain, intermittent pain, neuropathic pain and affective). However, investigators are required to sign a user agreement in order to access the SF-MPQ-2.
2. *Depression, anxiety, and health related quality of life* – There are already multiple validated measures already to assess this. They include the Endometriosis Health Profile Questionnaire (EHP-30) ^7^ or Short-Form Health Status survey, ^8^ but both require registration and/or payment. Beck Depression Inventory ^9^ and State Trait Anxiety Inventory ^10^ are also tools that could be used. Alternatively there are combined measures such as the Hospital Anxiety and Depression Scale ^11^ (however this is more for overall psychological distress rather than determining degree of anxiety or depression). Institutions may decide which scale to adopt.
3. *Menstrual history* –This includes details on age of menarche, cycle characteristics (including frequency, duration and amount) and changes in menstrual patterns over age ranges, as well as documenting hormone use.
4. *Fertility* – Information of the length of time a subject has tried to become pregnant without success, with subfertility being assessed as >6 months of trying. Information is gathered on fertility investigation and treatment. Pregnancy history and outcomes are also documented.
5. *Medical and surgical history* – This section also includes question on urinary symptoms as well as bowel symptoms (including questions from the Rome III ^12^ criteria irritable bowel syndrome module).
6. *Medication use* – Patterns and type of analgesia/ herbal supplements/ sleeping aid are documented in this segment.
7 *Personal information* - Ethnicity, age, BMI, (also lowest and highest weight since the age of 18yrs, somatotype and body shape by age range), level of education, smoking, exercise and alcohol consumption. Interestingly also questions on hair and eye colour looking at marking genetic subpopulations.

The third and fourth papers in this sequence dealt respectively with standard operating procedures for collection, processing and long term storage of fluid biospecimens ^3^ and tissue ^4^. These papers have less direct relevance to clinicians.

## Summary

The four papers are freely available for clinicians to obtain further information. It is hoped that implementation should be easily achievable as much that is required is standard practice. The project will ensure there is full documentation of patient demographics, history, care, surgical findings and specimen control enabling clinicians everyday gold standard care to be used in robust research studies and finally allowing for a significant sample size to be obtained and hopefully for firm recommendations to be able to made on patient care.
