# Supplementary material for: EPHect – the Endometriosis Phenome (and Biobanking) Harmonisation Project – may be very helpful for clinicians and the women they are treating
Source: F1000Res. 2017 Jan 5;6:14. [Version 1] doi: 10.12688/f1000research.9850.1 (PMC5428485; doi:10.12688/f1000research.9850.1)
Supplement: Peer review history

# Peer review: Referee response for version 1

**DOI:** 10.5256/f1000research.10620.r22139

**Published:** 2017-05-08

**Authors:** Maria Grazia Porpora

**Affiliations:** 1 Department of Gynecology, Obstetrics and Urology, University of Rome, Rome, Italy

The aim of this paper is to summarize four articles published by the World Endometriosis Research Foundation, aiming to set up the Endometriosis Phenome and Biobanking Harmonisation Project. This is a global initiative with the mission to develop a consensus on standardization and harmonization of phenotypic surgical/clinical data and biological sample collection methods in endometriosis management and research. It provides forms for collection of data related to surgery, clinical, and epidemiological phenotyping characteristics as well as standard operating procedures, processing, and long-term storage of biological samples from affected women. This project will allow data analysis of a large number of cases using the same forms worldwide, and the possibility of sharing the obtained information.

 This paper offers a clear and comprehensible recapitulation of the standard surgical forms and standard endometriosis patient questionnaires to facilitate their use by the clinicians in the clinical practice.

 Nevertheless, this paper does not analyze the limitations of the project such as the possible differences in resources and logistics among centers that could affect data collection and implementation of standard operating procedures. The extent and type of data collected and the effort to record data in an online database could be influenced by availability of time, local organizational structures and experience as well as motivation of the surgeon.

I have read this submission. I believe that I have an appropriate level of expertise to confirm that it is of an acceptable scientific standard.

# Peer review: Referee response for version 1

**DOI:** 10.5256/f1000research.10620.r21915

**Published:** 2017-04-18

**Authors:** Alan Lam

**Affiliations:** 1 Center for Advanced Reproductive Endosurgery, University of Sydney, St Leonards, Australia

This review article is a timely ‘call-to-arms’ to clinicians caring for women suffering from endometriosis world-wide to utilise the Endometriosis Phenome and Biobanking Harmonisation Project (EPHect).

 Published by the World Endometriosis Research Foundation (WERF) in 2014, EPHect provides a fundamental framework for collaborative international endometriosis research through global standardization of phenotypic data compilation and biological sample collection and storage.

 Concerned that the uptake of the EPHect has be sporadic and not uniform, the authors believe that the collection of “standardized detailed information”, using the standard surgical form (EPHect SSF) and standard endometriosis patient questionnaire (EPQ-S), should be ‘non-negotiable’ whenever surgery is undertaken for endometriosis.

 In practice, there are a number of challenges to the uptake of the EPHect which this review article has not addressed. Firstly, the time taken for busy clinicians and surgeons to complete the standard surgical form (SSF) is a potential impediment. Secondly, the completion of the standard endometriosis patient questionnaire (EPQ-S) or the minimum (EPG-M) by patient also requires motivation and time. Thirdly, the storage of the large amount of paper-based information is space-consuming. Fourthly, the transfer of information collected onto an electronic database is time-consuming, with privacy coding an issue. Finally, the storage, extraction, collation and analysis of data for the purpose of collaborative international research is likely going to be an expensive endeavour.

I have read this submission. I believe that I have an appropriate level of expertise to confirm that it is of an acceptable scientific standard.
